# Protective Effects of Membrane-Anchored and Secreted DNA Vaccines Encoding Fatty Acid-Binding Protein and Glutathione S-Transferase against *Schistosoma japonicum*

**DOI:** 10.1371/journal.pone.0086575

**Published:** 2014-01-23

**Authors:** Yaqin Tu, Yang Hu, Guorun Fan, Zhihao Chen, Lin Liu, Dandan Man, Shuojie Liu, Chengwu Tang, Yin Zhang, Wuxing Dai

**Affiliations:** 1 Department of Biochemistry and Molecular Biology, Tongji Medical College, Huazhong University of Science and Technology, Wuhan, Hubei, China; 2 Department of Otorhinolaryngology, Union Hospital of Tongji Medical College, Huazhong Science and Technology University, Wuhan, Hubei, China; Queensland Institute of Medical Research, Australia

## Abstract

In order to explore the high performance bivalent DNA-based vaccine against schistosomes, SjFABP and Sj26GST were selected and used to construct a vaccine. Two strategies were used to construct the bivalent DNA vaccine. In the first strategy, a plasmid encoding antigen in the secreted form was used, while in the other, a plasmid encoding a truncated form of SjFABP and Sj26GST targeted to the cell surface was used. Various parameters, including antibody and cytokine response, proliferation, histopathological examination, and characterization of T cell subsets were used to evaluate the type of immune response and the level of protection against challenge infection. Injection with secreted pIRES-sjFABP-sj26GST significantly increased the levels of antibody, splenocyte proliferation, and production of IFN-γ, compared with membrane-anchored groups. Analysis of splenic T cell subsets showed that the secreted vaccine significantly increased the percentage of CD3^+^CD4^+^ and CD3^+^CD8^+^ T cells. Liver immunopathology (size of liver granulomas) was significantly reduced in the secreted group compared with the membrane-anchored groups. Moreover, challenge experiments showed that the worm and egg burdens were significantly reduced in animals immunized with recombinant vaccines. Most importantly, secreted Sj26GST-SjFABP markedly enhanced protection, by reducing worm and egg burdens by 31.8% and 24.78%, respectively, while the membrane-anchored group decreased worm and egg burdens by 24.80% and 18.80%, respectively. Taken together, these findings suggest that the secretory vaccine is more promising than the membrane-anchored vaccine, and provides support for the development and application of this vaccine.

## Introduction

Schistosomiasis, a tropical disease that is caused by *Schistosoma japonicum*, is the most endemic and zoonotic disease in China, causing morbidity and mortality in almost 90 counties and affecting around 59 million individuals [1]. Although remarkable progress has been made in the chemotherapy treatment for schistosomiasis, problems such as reinfection, drug resistance, and radical cure are still a concern. New and effective therapies are required to treat the disease; however, the development of efficacious vaccines would help overcome the deficiencies of traditional *Schistosoma* snail-eradication methods and ineffective treatments.

DNA vaccines are promising when compared to other types of vaccines such as attenuated, subunit, and genetically engineered vaccines. They have low cost of production and high thermal stability, and are convenient to store. The World Health Organization (WHO) has recommended 6 major antigens, including membrane proteins, muscle components, and enzymes, as candidates for a more potent DNA vaccine for schistosomiasis. Among these antigens, the fatty acid-binding protein FABP (SjFABP) and glutathione S-transferase GST (Sj26GST) were shown to induce protective immunity in several laboratory studies [2], [3], [4]. Since parasites experience complicated life cycle stages and antigenic mutations to escape the host’s immunosurveillance, a single antigen is insufficient for inducing sufficient immune responses against schistosomes because of the relatively limited epitope. In comparison, multivalent DNA vaccines produce a variety of antigens with a large number of epitopes that can elicit a robust immune reaction, thus making them more potent and effective.

Vaccine-encoded protein antigens are either secreted or cell associated, with the antigen anchored on the cell surface [5]. Traditionally, secretory proteins are better vaccine candidates because they generally last longer, are likely to be stable, contain immune-related binding peptides, and are involved in the regulation of metabolic processes [6]. Excretory products of 6-day-old ex vivo larvae elicited strong immune responses and significant (P<0.05) protection against challenge infection in BALB/c mice [7]. Alternatively, studies on membrane proteins are gaining popularity. Using mice and macaque experimental models, Xavier et al. showed that a plasmid encoding a truncated form of the hepatitis C virus (HCV) E2 protein that is expressed on the cell surface is more immunogenic than a plasmid encoding intracellular E2 [8]. In addition, 2 surface-exposed tegument proteins of *S. mansoni* have shown efficacy as vaccines in a mouse model of schistosomiasis [9], [10]. Schistosome vaccine studies have not yet established whether secreted vaccines are less immunogenic than membrane-anchored vaccines.

In the present study, we selected SjFABP and Sj26GST as antigens and constructed 2 bivalent vaccines encoding either secretory proteins or membrane-anchored proteins to determine which one induced stronger immune responses and led to greater protective effects.

## Materials and Methods

### Ethics Statement

Animal experiments were performed in strict accordance with the National Institutes of Health “Guide for the Care and Use of Laboratory Animals” (NIH Publications No. 80–23, revised 1996), and all efforts were made to minimize suffering. All animal procedures were approved by the Tongji Medical College Committee on Animal Research, HUST (Permit Number: S270).

### Animals and Parasites

BALB/c male mice, 6–8 weeks old, were purchased from the Centers for Disease Control in Hubei Province. *S. japonicum*-infected *Oncomelania hupensis* snails (Chinese mainland strain) were purchased from Jiangsu Institute of Parasitic Diseases. Cercariae were collected from infected snails that were exposed to light. The quantity of cercariae was then assessed with a light microscope. Adult worms were harvested from cercariae-infected New Zealand rabbits by perfusion through the mesenteric vein 42 days after infection. Soluble worm antigen preparation (SWAP) was obtained by harvesting the soluble fraction from sonicated *S. japonicum* adult worms [11].

### DNA Vaccine Constructs

**Figure 1:**
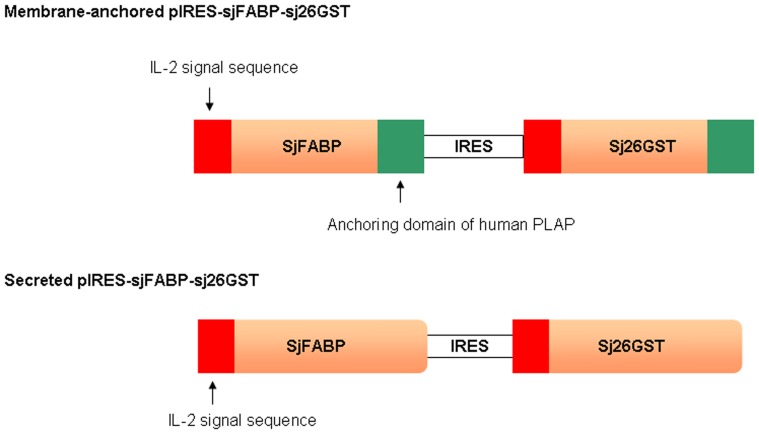
Schematic representation of secreted pIRES-sjFABP-sj26GST and membrane-anchored pIRES-sjFABP-sj26GST gene construction. SjFABP and Sj26GST were chosen as the antigens and pVAC was used as the vector. SjFABP and Sj26GST were modified by addition of 2 coding sequences: A 23-amino acid signal peptide from human IL-2 was added upstream and a membrane-anchored sequence containing 32 amino acids of the carboxyl terminal of human placental alkaline phosphatase (PLAP) was added downstream. The 2 modified genes were then subcloned into a pIRES eukaryotic expression vector, which is a new-generation multigenic vector with 2 open reading frames that enables each product of transcription to be translated independently. The antigens expressed in the recombinant vaccine were then anchored to the cell membrane. The absence of the membrane-anchored sequence downstream led to the synthesis of a secretory antigen.

The membrane-anchored pIRES-sjFABP-sj26GST DNA vaccine encoding a fatty acid-binding protein and the 26-kDa *S. japonicum* glutathione S-transferase gene was previously constructed in our laboratory [12]. The secreted pIRES-sjFABP-sj26GST plasmid was constructed using the same protocol as the one used for the membrane-anchored vaccine, except that the placental alkaline phosphatase (PLAP) membrane-anchored sequence at the 3′-end of the designed primer was removed (Fig. 1). Constructs were confirmed by restriction analysis and sequencing. The expression of the above DNA vaccines was also tested by western blot and by indirect immunofluorescence. Maxipreps of the plasmids were prepared using the EndoFree Maxi Plasmid Kit (Qiagen, Germany), as described in the manufacturer’s protocol.

### Immunization and Challenge Protocol

Three different groups of mice (10 mice in each group) were inoculated intramuscularly in the quadriceps femoris muscle with 100 µg of the plasmids harboring secreted pIRES-sjFABP-sj26GST or membrane-anchored pIRES-sjFABP-sj26GST on day 1 followed by a boost on day 21 and 42. Animals injected with an empty pIRES vector were used as the control. Blood was collected from the tail vein prior to immunization. Two weeks after the final vaccination, mice were sacrificed for characterization of their cellular and humoral immune responses.

**Figure 2:**
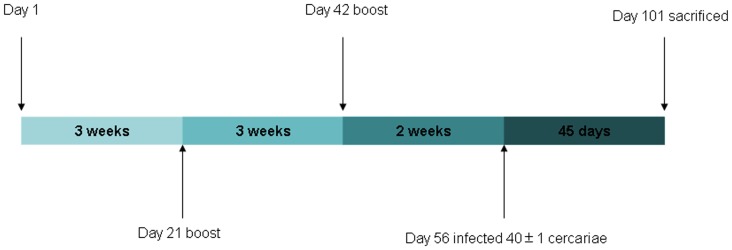
Mouse immunization protocol. Three groups of mice (10 mice in each group) were subcutaneously injected with 100 µg secreted pIRES-sjFABP-sj26GST, membrane-anchored pIRES-sjFABP-sj26GST, and pIRES plasmids on day 1 and boosted on day 21 and 42. Two weeks after the last boost all mice were challenged with 40±1 cercariae by abdominal skin penetration. Forty-five days post challenge, the mice were sacrificed.

For the vaccination challenge trial, mice were divided into 3 groups of 10 mice per group. Each mouse was intramuscularly injected with 100 µg of secreted pIRES-sjFABP-sj26GST, membrane-anchored pIRES-sjFABP-sj26GST, or pIRES. Immunization was repeated 3 times at 21-day intervals. Two weeks after the final vaccination, all mice from each group were challenged with 40±1 *S. japonicum* cercariae by abdominal skin penetration [13] (Fig. 2).

### Antibody Assays

To evaluate the IgG antibodies, sera from 10 vaccinated mice from each group were tested using a mouse serum IgG ELISA kit (American 4ADI Company) as described previously [14]. Briefly, the 96-well flat-bottomed plates (Corning, USA) were coated with 2.5 µg SWAP in coating buffer and stored at 4°C overnight. The plates were then washed three times with washing buffer and then blocked with blocking buffer for 1h at room temperature. After blocking, the plates were washed three times and then incubated with diluted serum for detection of IgG. The reaction was then terminated and the optical density (OD) was read at 450 nm.

### Splenocyte Proliferation Assay

Splenocytes were harvested from immunized (secreted pIRES-sjFABP-sj26GST and membrane-anchored pIRES-sjFABP-sj26GST) or control (vector immunized) mice 2 weeks after the last inoculation. Cells were seeded in triplicate in 96-well flat-bottom plates at 2×10^5^ cells/well in RPMI-1640 supplemented with 10% fetal bovine serum and 1% antibiotics (100 U/ml penicillin G and 100 µg/ml streptomycin). Cells derived from each experimental group were cultured in the presence of 2.5 µg SWAP (stimulated) or in the absence of SWAP (unstimulated controls). After incubation for 48 h at 37°C with 5% CO_2_, the supernatant was removed and then 100 µl of DMEM and 10 µl MTT (MTT, 5 mg/ml; Sigma) were added to each well for 6 h. Following incubation, 100 µl of dimethylsulfoxide (DMSO) was added to solubilize the sediment and finally the absorbance was read at 570 nm. The stimulation index (SI) was calculated as the ratio of the A_570_ nm absorbance of stimulated cells to that of unstimulated cells.

### Cytokine Detection

To evaluate cytokine production, single-cell splenocyte suspensions were adjusted to a concentration of 5×10^6^/ml and cultured in the presence of 2.5 µg/ml SWAP. Cell-free supernatants were collected and assayed for interleukin-2 (IL-2) and interleukin-4 (IL-4) at 48 h, and for interleukin-10 (IL-10) and interferon gamma (IFN-γ) at 72 h using a Th1/Th2 cytokine ELISA kit (eBioscience, CA) according to the manufacturer’s instructions.

### Detection and Analysis of Subsets of Spleen T Lymphocytes

Lymphocytes were harvested from immunized (membrane-anchored pIRES-sjFABP-sj26GST and secreted pIRES-sjFABP-sj26GST) or control (pIRES) mice 14 days after the last immunization. Cells were resuspended in FACS buffer, and the final concentration of each sample was adjusted to 1×10^8^/ml. Each sample was stained with antibodies to various monoclonal T cell markers (BioLegend, USA) as described in the manufacturer’s protocol. Briefly, 1 µl of PE-conjugated anti-mouse CD4, 1 µl FITC-conjugated anti-mouse CD3, and 1 µl APC-conjugated anti-mouse CD8 were sequentially added into the samples before the mixture was incubated in the dark at 4°C for 30 min. After centrifugation, the supernatant was discarded and the cells were washed twice with cold PBS, before they were resuspended in 100 µl PBS. The samples were then examined using a FACS Calibur flow cytometer (BioLegend, CA) and the frequency of the CD3^+^CD4^+^ and CD3^+^CD8^+^ cells among total splenocytes were acquired and analyzed with CellQuest software.

### Measurement of NO Production in Peritoneal Macrophages

Macrophages were acquired by lavage of the peritoneal cavity with wash medium. The harvested macrophages were then incubated in 24-well flat-bottom plates (Corning, USA) with 2 ml RPMI-1640 containing 10% fetal bovine serum (Life Technologies, USA) for 2 h. Nonadherent cells were removed by washing after a 2-h incubation.

NO was detected as described in the manufacturer’s protocol by using Griess reagent (Promega, USA). In brief, 100 µl of the culture supernatant was added to wells in triplicate, followed by sequential addition of Griess Reagent I to each well. Finally, Griess Reagent II was added to the wells and the plates were then incubated until a purple or magenta color was observed. The absorbance was then read at 550 nm within 30 min.

### Evaluation of Worm and Egg Burden in the Liver

Forty-five days after challenge, adult worms were collected from livers and were harvested from each group by perfusing the mesenteric vein. To evaluate the anti-schistosoma protective activity of the vaccines, the livers were weighed and digested with 5% KOH solution overnight at 37°C. The egg and worm reduction rates were then calculated using the following formulae: Egg reduction rate (%) = (eggs per gram in control group − eggs per gram in experimental group)/eggs per gram in control group×100%; worm reduction rate (%) = (average number of worms in control group − average number of worms in experimental group)/average number of worms in experimental group×100%.

### Histological Studies

After portal perfusion, the remaining liver lobe from each mouse was dissected and immediately fixed in 10% buffered formalin after washing with PBS. Liver sections were embedded in paraffin and cut into 4 µm sections. The sections were then examined by light microscopy after standard hematoxylin and eosin (H&E) staining for visualization of cellular changes. To more accurately reflect the true shape and dimensions of the granulomas, only those containing clearly identifiable eggs at the center were selected. The area of liver egg granulomas was measured using computer-assisted morphometric software (Image-Pro Plus software 6.0). For each specimen, at least 3 non-continuous slides were measured and the mean values obtained from 10 mice from each group were used for statistical analysis.

### Statistical Analysis

Data are expressed as mean ± standard deviation (SD).Values for worm and egg burdens, antibody levels, cytokine levels, stimulating index, area of liver granulomas and NO levels were compared by One-Way ANOVA using SPSS 18.0 software (SPSS Inc. Chicago, IL, USA). In all analyses, a P value of less than 0.05 was considered statistically significant.

## Results

### Histologic Evaluation of Granuloma Formation

**Figure 3:**
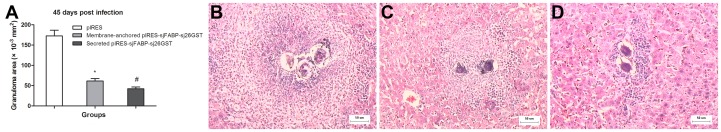
Reduction of hepatic granulomatous inflammation in the secreted vaccine group. (A) The granuloma area was calculated from isolated granulomas 45 days after infection (n = 10 for each group).*P<0.05, versus the control group; ^#^P<0.05, versus the membrane-anchored pIRES-sjFABP-sj26GST group. (B) Liver sections from the control (left panels), membrane-anchored (middle panels), and secreted groups (right panels) were examined by H&E staining.

Control, membrane-anchored, and secreted vaccine-treated mice were sacrificed, and morphological and histopathological differences in livers were evaluated. In the control group, the liver cells that had trapped the mature eggs were severely destroyed, and a large amount of extracellular matrix deposition and heavy inflammatory cell infiltration were observed around the eggs. The DNA-vaccinated groups, particularly those receiving secreted vaccine treatment, demonstrated obvious amelioration in egg granuloma formation and less inflammatory cell infiltration in egg granulomas (Fig. 3B). The extent of hepatic granulomatous inflammation around the schistosome eggs was measured by computer-assisted morphometric analysis. Infected livers from the membrane-anchored group developed large liver granulomas (mean cross-sectional area, 61.53±32.48 mm^2^×10^−3^), while these granulomas were significantly ameliorated in the secreted vaccine-treated group (42.50±22.87 mm^2^×10^−3^; Fig. 3A; P<0.05).

### Antibody Responses in DNA Vaccinated Mice

**Figure 4:**
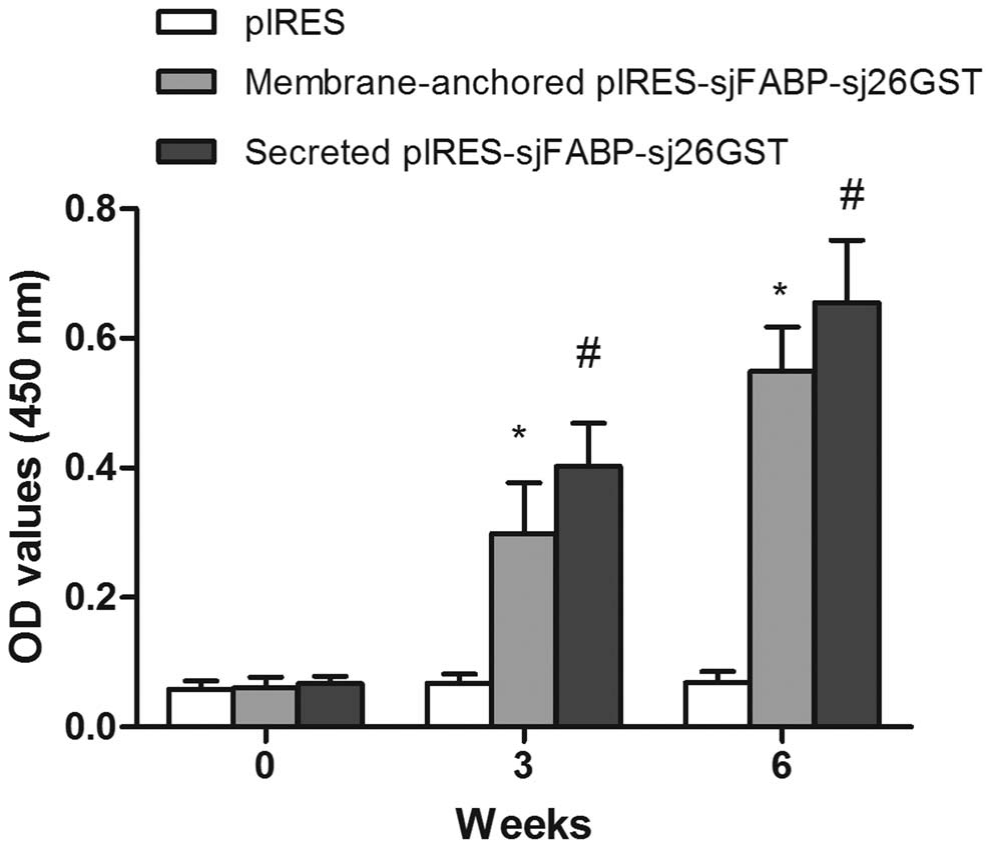
Analysis of the serological immune response to the vaccine. Total IgG antibody responses in mice injected with secreted pIRES-sjFABP-sj26GST, membrane-anchored pIRES-sjFABP-sj26GST, and pIRES as control. Absorbance values (450 nm) are shown as mean ± SD of 10 mice in each group. Results are statistically significant (P<0.05) when compared to the pIRES group: *; from the membrane-anchored group: ^#^.

Serum samples of each mouse collected prevaccination and 3 weeks after each injection were analyzed separately for total IgG antibodies by ELISA. The average antibody titers of each group against specific antigens are shown in Fig. 4. Vaccination with membrane-anchored or secreted pIRES-sjFABP-sj26GST induced significant and progressive increases in total IgG responses against SWAP (P<0.05), compared with the control group injected with pIRES. Notably, injection of secreted pIRES-sjFABP-sj26GST significantly enhanced IgG responses after the second vaccination (P<0.05), compared with vaccination with membrane-anchored pIRES-sjFABP-sj26GST.

### Splenocyte Proliferation

To determine the effects of the vaccine on the immune response following antigen-specific stimulation, splenocyte proliferation was assessed 2 weeks after the last immunization. As shown in Table 1, the 2 groups immunized with membrane-anchored and secreted pIRES-sjFABP-sj26GST showed significant induction of lymphocyte responses, compared with the control group (P<0.05). Furthermore, the secretory vaccine group produced more robust lymphocyte responses than did the membrane-anchored vaccine group (P<0.05).

**Table 1:** Splenocyte proliferative responses in mice immunized with the membrane-anchored vaccine or the secreted DNA vaccine.

Group	Mean stimulation index±S.D.
pIRES	1.17±0.18
Membrane-anchored pIRES-sjFABP-sj26GST	1.77±0.24*
Secreted pIRES-sjFABP-sj26GST	2.63±0.27**

The lymphocyte proliferative assay was performed 2 weeks after the last vaccination. Data represent the mean ± SD.

* Results were statistically significant when compared to the PIRES group (P<0.05).

** Results were statistically significant when compared to the pIRES-sjFABP-sj26GST membrane-anchored group (P<0.05).

### T Lymphocyte Subset Analysis

**Figure 5:**
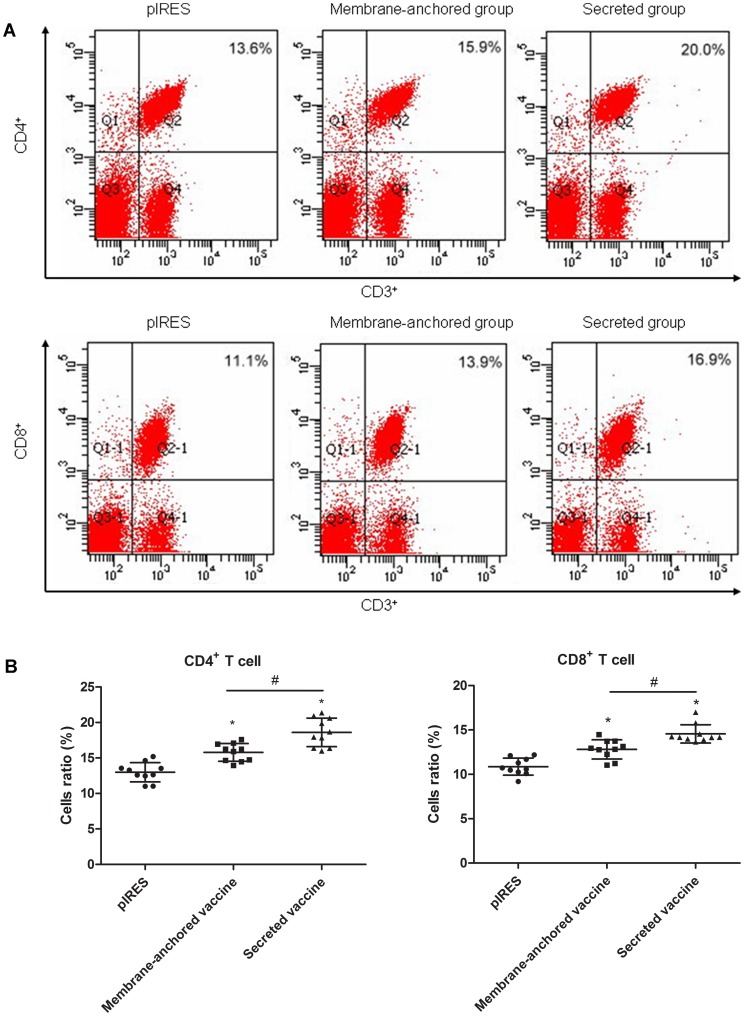
Percentage of CD3^+^CD4^+^ and CD3^+^CD8^+^ in total splenocytes as detected by flow cytometry. A: Representative flow cytometry data demonstrating the percentage of CD3^+^CD4^+^ and CD3^+^CD8^+^ cells in total splenocytes from each of the treatment group. B: Flow cytometry demonstrating that the secreted vaccine significantly increased the percentage of CD3^+^CD4^+^ and CD3^+^CD8^+^ T cells in total splenocytes. *P<0.05, versus the control group; ^#^P<0.05, versus the membrane-anchored pIRES-sjFABP-sj26GST group.

Two weeks after the last immunization, T lymphocyte subsets in each group were analyzed by flow cytometry (Fig. 5). The secreted and membrane-anchored pIRES-sjFABP-sj26GST groups both showed higher percentage of CD3^+^CD4^+^ T lymphocytes than the control group did (P<0.05), while the secretory vaccine group showed induction of higher proportions of CD3^+^CD4^+^ and CD3^+^CD8^+^T lymphocytes than did the membrane-anchored vaccine group in the spleen (P<0.05).

### Th1 and Th2 Cytokine Measurement from Splenocyte Culture Supernatants

**Figure 6:**
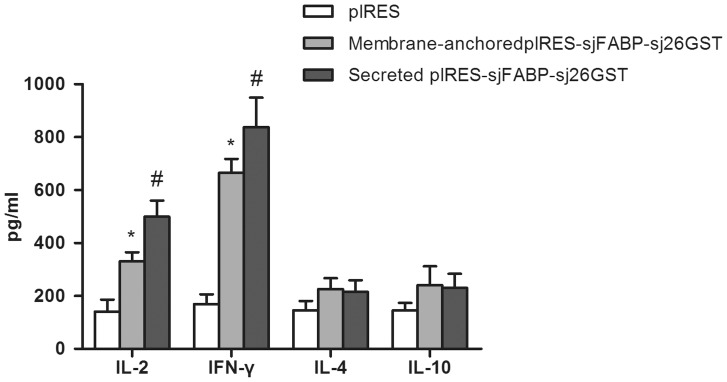
Levels of cytokine production from splenocytes after SWAP stimulation in Groups of mice were injected with secreted pIRES-sjFABP-sj26GST, membrane-anchored pIRES-sjFABP-sj26GST, and pIRES as control. Data from 3 experiments are expressed as the mean ± SD. Statistically significant differences (P<0.05) are indicated by *compared with the control group, or^ #^ compared with the membrane-anchored group.

The cellular immunity in immunized mice was indirectly evaluated by measuring cytokine levels in the supernatants from the cultures of SWAP-stimulated spleen cells. As shown in Fig. 6, the levels of all 4 cytokines were higher in the secretory vaccine group and the membrane-anchored vaccine group than in the pIRES vector group. The production of IFN-γ and IL-2 (Th1-type) was more robust in the secretory vaccine group than in the membrane-anchored vaccine group (P<0.05). However, no significant difference was observed in the levels of IL-4 and IL-10 between the 2 experimental groups. We also measured the serum levels of Th1/Th2 cytokines. Mice in the secreted vaccine-administered group had significantly higher concentrations of IL-2 and IFN-γ compared with the membrane-anchored vaccine-administered group (Figure S1), which correlates well with our in vitro results. This profile of cytokine secretion suggests that the secretory antigen enhances the induction of immune responses by promoting a Th1-dominant response.

### Assessment of NO Production in Peritoneal Macrophages

The peritoneal cavity of each mouse was washed 2 weeks after the last immunization, and macrophages were harvested. The NO in the activated macrophages was then quantified as described in the Methods section. As shown in Table 2, injection of membrane-anchored pIRES-sjFABP-sj26GST and secreted pIRES-sjFABP-sj26GST significantly augmented the production of NO, compared with the control group. Furthermore, the secretory vaccine group had slightly elevated NO levels when compared to the membrane-anchored vaccine group (P<0.05).

**Table 2:** Determination of NO in macrophages.

Groups	No. of mice	NO (nmol/ml)
pIRES	10	184.12±11.05
Membrane-anchored pIRES-sjFABP-sj26GST	10	262.19±15.10*
Secreted pIRES-sjFABP-sj26GST	10	280.47±13.08**

* Results were statistically significant when compared to the pIRES group (P<0.05).

** Results were statistically significant when compared to the pIRES-sjFABP-sj26GST membrane-anchored group (P<0.05).

### Secreted DNA Vaccine Induces Greater Immune Protection against *S. japonicum* Challenge

Infected mice were euthanized 45 days after infection in order to determine the adult worm reduction and egg reduction rates in the liver. As shown in Table 3, mice injected with membrane-anchored pIRES-sjFABP-sj26GST or secreted pIRES-sjFABP-sj26GST showed a significant worm reduction and egg reduction rate in the liver, compared with the control group (P<0.05). Most importantly, secreted vaccines were superior to membrane-anchored vaccines at enhancing immune protection with a worm reduction rate of 31.8% and an egg reduction rate of 24.78%. In addition, the statistical significance in the worm reduction rate was more remarkable than that in egg reduction rate, thus illustrating that the secreted vaccines played a much more important role in eliminating worms than eggs.

**Table 3:** Protective effects of immunization with plasmid membrane-anchored pIRES-sjFABP-sj26GST, secreted pIRES-sjFABP-sj26GST, and pIRES following *S. japonicum* challenge in BALB/c mice.

Group	Worm burden	Worm reductionrate (%)	Average number of eggsper gram liver tissue (×10^4^)	Liver egg reduction rate (%)
pIRES	25.8±2.86	/	3.4±0.1557	/
Membrane-anchored pIRES-sjFABP-sj26GST	19.4±1.5776	24.80% *	2.7614±0.076	18.80% *
Secretory pIRES-sjFABP-sj26GST	17.6±1.646	31.80% **	2.558±0.154	24.78% **

All results are presented as mean ± SD. The mice were vaccinated with 100 µg membrane-anchored pIRES-sjFABP-sj26GST, secreted pIRES-sjFABP-sj26GST, and pIRES plasmid twice with a 3-week interval. Two weeks after final boosting, all groups were infected with 40±1 *S. japonicum* cercariae. Mice were euthanized 45 days after challenge at which point adult worm burdens and egg numbers in feces and liver were compared among the 3 groups.

* Results were statistically significant when compared to the PIRES group (P<0.05).

** Results were statistically significant when compared to the pIRES-sjFABP-sj26GST membrane-anchored group (P<0.05).

## Discussion

Over the past 50 years, there has been a concerted effort to develop vaccines against schistosoma. However, due to the complicated life cycle of *Schistosoma japonicum* and the sophisticated strategies it uses to escape the host immune assault, results from dedicated vaccine programs have been disappointing [15]. To our knowledge, no studies have been conducted to compare the immunogenicity between vaccines encoding secretory and membrane-anchored proteins in terms of *S. japonicum*. Therefore, increased understanding of the differences in immunity that these 2 types of vaccines induce is vital for optimizing vaccine design and delivery.

In the present study, 3 injections of the experimental DNA constructs at 3-week intervals induced a significantly higher lymphocyte proliferative response, and IgG antibody response against *S.japonicum* antigens than the control. In addition, these responses were enhanced by intramuscular injection of the secreted vaccine. Our results reveal that secretory antigens can augment both humoral and cell-mediated immune responses in vaccine-induced immunity against pathogens and can enhance the proliferative response of immune cells. These results are in agreement with those reported by other researchers using different immunization schemes [16], [17].

It has been previously shown that in putative resistant populations, both Th1 and Th2 type cytokines are induced and detected after antigen stimulation [18], whilst in chronically infected individuals, a Th2 type response is more dominant [19]. Th1 cells are known to be responsible for the activation of cell-mediated responses associated with IFN-γ, IL-2 and IL-12 [20], [21]. IFN-γ is thought to be pivotal in resisting infections from schistosomiasis [22]. Consistent with the findings of many other studies, our results show that the secreted vaccine, when compared with the membrane-anchored vaccine, increased the Th1 type immune response with significant production of IFN-γ and low levels of IL-4, indicating a stronger Th1 response [3], [23], [24].

T cell subsets are important for the host’s immune defense against infection. Granuloma formation and the resulting pathology are dependent on and are mediated by CD4^+^ T cells responding to egg antigens [25]. The immune response to schistosomes at the early stage of infection increases the number of CD4^+^ Th1 cells and CD8^+^ T cells and leads to pathologic liver changes. When pathologic changes occur in the liver, the host immune response is modulated by CD4^+^ helper Th2 T cells and suppressive CD8^+^ T cells [26]. Our flow cytometry (FCM) results clearly showed that the percentages of CD3^+^CD4^+^ and CD3^+^CD8^+^ T cells in the secreted and membrane-anchored vaccine groups increased modestly, indicating that the vaccine can enhance T helper cell-mediated immunization and cytotoxic T lymphocyte (CTL) responses. Although there was no statistically significant difference in the percentages of CD4^+^ and CD8^+^ T cells among the CD3^+^ cells between the experimental and control groups (Figure S2A), the number of CD3^+^ cells among the total splenocytes were prominently elevated in the vaccinated groups, particularly in the secretory vaccine group (Figure S2B; P<0.05). The dynamic equilibrium of the CD4^+^ and CD8^+^ T cell ratio determines the immune status and immune competence of the host [27]. An increasing CD4^+/^CD8^+^ ratio was observed in the secreted group although there was no significant difference between the secretory and membrane-anchored groups in terms of the ratio (data not shown). These data are consistent with those of a recent study showing that declining CD4^+/^CD8^+^ ratios are more likely to result in a weakened immune response [25].

The granulomatous reaction plays an important role in protecting host tissues by sequestering antigens released by the parasite eggs. Various complex immune modulatory mechanisms take part in the granuloma-formation process [28]. Granuloma formation is the result of a host adaptive immune response mediated by CD4^+^ T cells specific for schistosome egg antigens (SEAs) [29], [30] that damages hepatocytes and destroys the normal histological structure of the liver (Fig. 3B). As expected, the granulomatous response in liver was suppressed in the secreted vaccine group compared with other groups as measured by the granuloma cross-sectional area. This phenomenon may be due to the larger proportion of CD4^+^ T cells detected in the secreted vaccinated mice [31].

Macrophages can be stimulated to produce a number of chemokines, cytokines, and antioxidants such as NO, for the primary protection of the host after infection with *S. japonicum* [32]. Our study showed that the production of NO was significantly increased in the macrophages harvested from mice that had been immunized with secreted SjFABP-Sj26GST vaccine, indicating that the secreted vaccine had a better ability to eliminate infectious pathogens. The worm and egg reduction rates serve as the “gold standard” of vaccine protection efficacy against schistosoma. In our current studies, results from the parasite challenge experiments consistently demonstrated that the secreted vaccine provided a higher level of protection than the membrane-anchored vaccine did, as measured by its ability to induce higher levels of worm and egg reduction rates in the liver.

Taken together, these data show that both secretory DNA vaccines and membrane-anchored vaccines preferentially promote Th1 cell-mediated immune responses. In addition, the secreted vaccines were more efficacious than the membrane-anchored vaccines when various measures of immunity, such as antibody titer, T lymphocyte proliferative responses, and protection against infection, were evaluated. The immunogenicity of proteins/antigens is known to be partly dependent on whether the antigens can be sufficiently exposed to and recognized by the immune system. The secretory proteins were expressed at high concentrations in the body fluid and thus interacted more efficiently with immune effectors. Membrane-anchored vaccines, on the other hand, were latent and almost inaccessible to the host immune system [7]. When exposed to host tissues, the secreted form of *S. japonicum* antigens can not only stimulates the innate immune system, but also modulates various host immune responses. Thus, the specific antibody and leukocyte-derived inflammatory cytokines can generate immune effectors that track, target, surround, and pursue the migrating larvae [7]. Of note, secreted antigens also induce memory effector cells during vaccination that can maintain long-term immune surveillance, while the membrane-anchored antigens seem to lack this ability [33]. In conclusion, the findings of the present study are novel and indicate that secreted vaccines may serve as an effective strategy for the immunoprophylaxis of *S. japonicum*,as well as provide a basis for the development of secreted vaccines against schistosoma.

## Supporting Information

Figure S1 **Levels of cytokine production in vivo.** Two weeks after the last immunization, serum was isolated and assayed for IL-2, IL-10, IL-4, and IFN-γ production. Data from 3 experiments are expressed as the mean ± SD. Statistically significant differences (P<0.01) are indicated by **compared with the membrane-anchored group.
(TIF)

Figure S2 **Percentages of CD4+ and CD8+ cells among the CD3+ cells detected by flow cytometry.** A: The ratio of CD3^+^CD4^+^/CD3^+^ and CD3^+^CD8^+^/CD3^+^. There was no statistically significant difference in the percentages of CD4^+^ and CD8^+^ T cells among the CD3^+^ cells between the experimental and control groups. B: Numbers of CD3^+^ cells among the total splenocytes. Flow cytometry showed that the number of CD3^+^ cells among the total splenocytes increased significantly in the vaccinated groups. Values represent means ± SD. N = 10 per group. *P<0.05, versus the control group; ^#^P<0.05, versus the membrane-anchored pIRES-sjFABP-sj26GST group.
(TIF)
