# Supplementary figures and images for: Protective Effects of Membrane-Anchored and Secreted DNA Vaccines Encoding Fatty Acid-Binding Protein and Glutathione S-Transferase against Schistosoma japonicum
Source: PLoS One. 2014 Jan 23;9(1):e86575. doi: 10.1371/journal.pone.0086575 (PMC3900569; doi:10.1371/journal.pone.0086575)

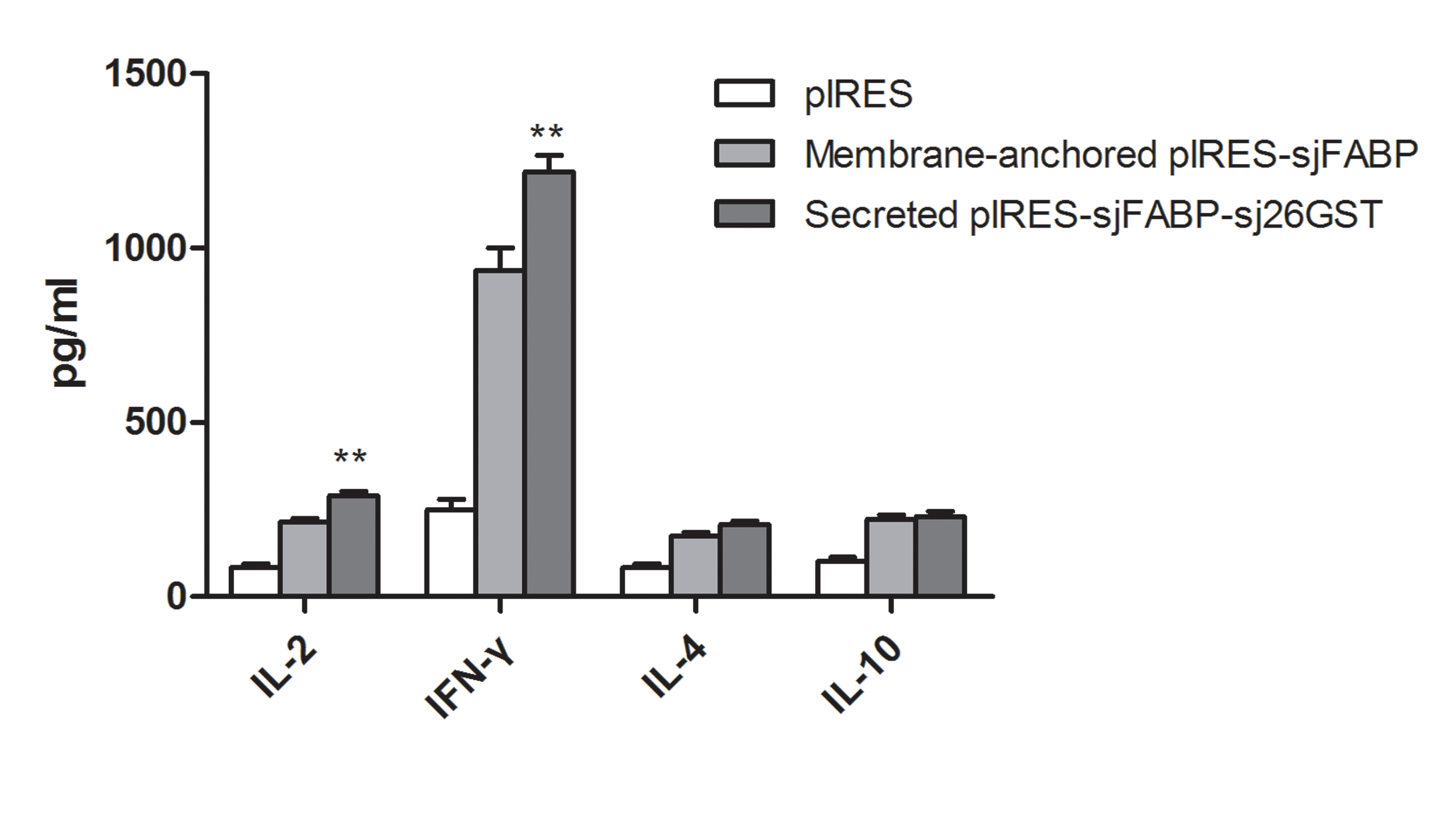

Supplement: Figure S1 — Levels of cytokine production in vivo. Two weeks after the last immunization, serum was isolated and assayed for IL-2, IL-10, IL-4, and IFN-γ production. Data from 3 experiments are expressed as the mean ± SD. Statistically significant differences (P<0.01) are indicated by **compared with the membrane-anchored group. (TIF) [file pone.0086575.s001.tif]

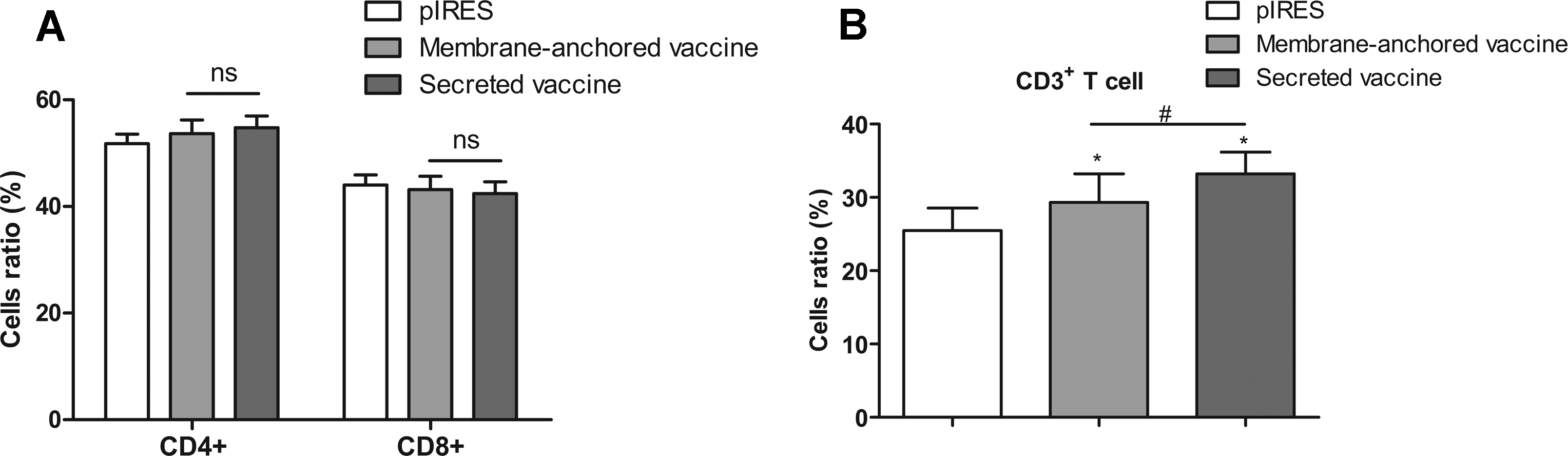

Supplement: Figure S2 — Percentages of CD4+ and CD8+ cells among the CD3+ cells detected by flow cytometry. A: The ratio of CD3+CD4+/CD3+ and CD3+CD8+/CD3+. There was no statistically significant difference in the percentages of CD4+ and CD8+ T cells among the CD3+ cells between the experimental and control groups. B: Numbers of CD3+ cells among the total splenocytes. Flow cytometry showed that the number of CD3+ cells among the total splenocytes increased significantly in the vaccinated groups. Values represent means ± SD. N = 10 per group. *P<0.05, versus the control group; #P<0.05, versus the membrane-anchored pIRES-sjFABP-sj26GST group. (TIF) [file pone.0086575.s002.tif]
